# Effect of Magnesium as Substitute Material in Enzyme-Mediated Calcite Precipitation for Soil-Improvement Technique

**DOI:** 10.3389/fbioe.2016.00037

**Published:** 2016-05-04

**Authors:** Heriansyah Putra, Hideaki Yasuhara, Naoki Kinoshita, Debendra Neupane, Chih-Wei Lu

**Affiliations:** ^1^Disaster Mitigation for Asian Students, Graduate School of Science and Engineering, Ehime University, Matsuyama, Japan; ^2^Engineering Faculty, Jambi University, Jambi, Indonesia; ^3^Department of Civil and Environmental Engineering, Ehime University, Matsuyama, Japan; ^4^Penta – Ocean Construction Co. Ltd, Tokyo, Japan; ^5^Department of Construction Engineering, National Kaohsiung First University of Technology, Kaohsiung, Taiwan

**Keywords:** EMCP, carbonate, magnesium chloride, delaying agent, soil improvement

## Abstract

The optimization of enzyme-mediated calcite precipitation was evaluated as a soil-improvement technique. In our previous works, purified urease was utilized to bio-catalyze the hydrolysis of urea, which causes the supplied Ca^2+^ to precipitate with $\text{C}{\text{O}}_{3}^{\text{2}-}$ as calcium carbonate. In the present work, magnesium chloride was newly added to the injecting solutions to delay the reaction rate and to enhance the amount of carbonate precipitation. Soil specimens were prepared in PVC cylinders and treated with concentration-controlled solutions composed of urea, urease, calcium, and magnesium chloride. The mechanical properties of the treated soil specimens were examined through unconfined compressive strength (UCS) tests. A precipitation ratio of the carbonate up to 90% of the maximum theoretical precipitation was achieved by adding a small amount of magnesium chloride. Adding magnesium chloride as a delaying agent was indeed found to reduce the reaction rate of the precipitation, which may increase the volume of the treated soil if used in real fields because of the slower precipitation rate and the resulting higher injectivity. A mineralogical analysis revealed that magnesium chloride decreases the crystal size of the precipitated materials and that another carbonate of aragonite is newly formed. Mechanical test results indicated that carbonate precipitates within the soils and brings about a significant improvement in strength. A maximum UCS of 0.6 MPa was obtained from the treated samples.

## Introduction

Bio-chemical grouting, as a ground-improvement method, has been studied for its various possible applications, such as the preservation of limestone monuments (Al-Thawadi, 2011), the plugging up of the pores in oil recovery reservoir rocks and the removal of contaminants in groundwater systems (Nemati et al., 2005), the reparation of cracks in concrete (De Muynck et al., 2008), the reduction of the swelling potential of clayey soil and the mitigation of the liquefaction potential of soil (Ivanov and Chu, 2008; Akiyama and Kawasaki, 2012; Putra et al., 2015), and the control of and the improvement in the soil permeability (Whiffin et al., 2007; van Paassen et al., 2009; DeJong et al., 2010; Harkes et al., 2010; Yasuhara et al., 2012). Thus far, most studies on enzymatic calcite grouting have used bacterial cells containing urease, e.g., *Sporosarcina pasteurii*, to dissociate urea into ammonium and carbonate ions (Whiffin, 2004; DeJong et al., 2006; Whiffin et al., 2007; van Paassen et al., 2010).

Microbially induced calcite precipitation (MICP) may be one of the promising bio-mediated soil-improvement techniques (DeJong et al., 2011; Martinez et al., 2013). MICP has been studied extensively for its potential as a novel soil-improvement technique. The increase in compressive strength ranges from 0.2 to 12 MPa depending on the amount of precipitated calcite (van Paassen et al., 2010).

Enzyme-mediated calcite precipitation (EMCP) (Yasuhara et al., 2011, 2012; Neupane et al., 2013) may be an alternative method for improving soil properties. An enzyme reagent mixed solution (i.e., CaCl_2_–urea–urease solution), which produces the precipitated calcite after the chemical reaction, is injected into the soil. The precipitated calcite may provide bridges between the grains of sand, restricting their movement, and hence, improving the stiffness and the strength of the soil (Yasuhara et al., 2011). For instance, the efficacy of 1–2 g/L of the enzyme with the activity of 2950 U/g was evaluated in our previous works (Neupane et al., 2013; Putra et al., 2015). A precipitation ratio of calcite of up to 70% was obtained using a small amount of a urease and reagent solution. The amount of precipitated calcite varied from 1.5 to 6.0% of the sand weight on the inner spherical portion, with a diameter of 30 cm, and the corresponding reduction in porosity ranged from 2 to 7%. The amount of precipitated calcite may be enough to modify the mechanical properties of sandy soil (Neupane et al., 2013). The treated sand displays sufficient strength as a grouting material to counteract soil liquefaction (Putra et al., 2015). However, the uniform distribution of calcite within a large domain has not yet been achieved. The precipitation rate may have a remarkable influence on the treatment area (Neupane et al., 2015).

A study on the carbonation rates of calcite has been reported by Apriliani et al. (2012). It was concluded in their study that the addition of magnesium to the inorganic carbonation process delayed the carbonate precipitation rate, modified the structure and the size of the precipitated crystals, and generated dolomite and magnesium carbonate (Apriliani et al., 2012). The concentration of Mg^2+^ ions influenced the morphology of the CaCO_3_ polymorphs, and the precipitated calcite was seen to possibly progress from angular to spherical as the Mg^2+^ ions increased (Boyd, 2012). The carbonation process described in the previous works was investigated inorganically, but it may also be examined in organic processes, such as MICP and EMCP.

In this work, various amounts of magnesium were used to examine the rate and the magnitude of carbonate precipitation when added to grouting materials, i.e., CaCl_2_–urea–urease solution (Yasuhara et al., 2011, 2012; Neupane et al., 2013, 2015). Moreover, the effects of the added magnesium exerted on the size and the structure of the precipitated crystals and the formation of other carbonate minerals (e.g., dolomite, magnesite, and aragonite) were also evaluated. The optimal combination of reagents was fixed by test-tube tests and then utilized to improve small-scale specimens. The microstructures of the precipitated carbonates were examined by X-ray powder diffraction (XRD) and scanning electron microscopy (SEM) to assess the effects of magnesium on the formation of carbonates. Unconfined compressive strength (UCS) tests were also performed to evaluate the improved mechanical properties of the treated specimens by the presence of the magnesium. Finally, by comparing the relation between the UCS and the amount of carbonate precipitated within the treated specimens obtained in this work, with those obtained from the literature, the influence of the added magnesium was explicitly investigated.

## Materials and Methods

### Materials

**Figure 1:**
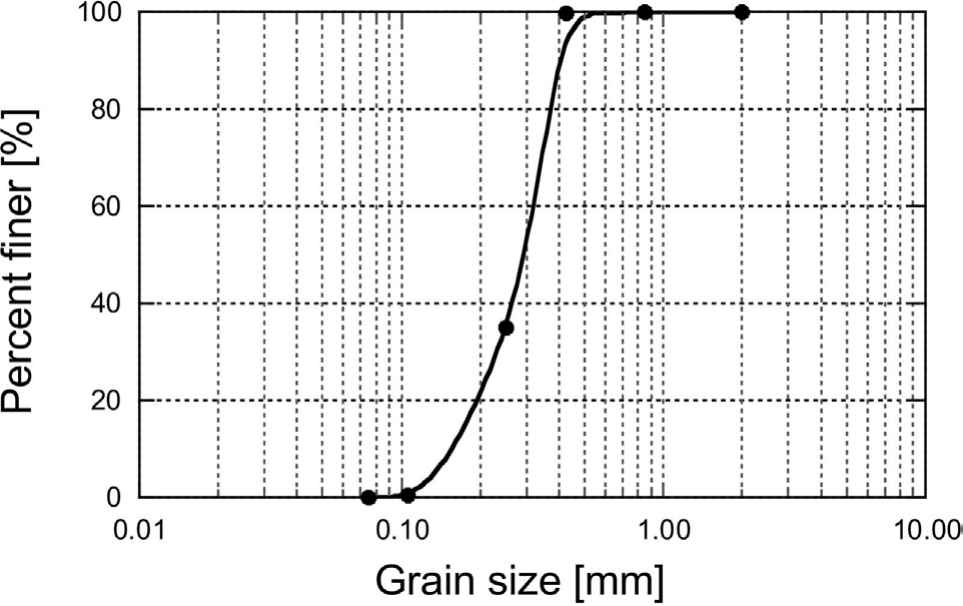
**Grain size distribution curve of sand**.

Urea, CaCl_2_, and MgCl_2_, with claimed purity levels >95.0%, were obtained from Kanto Chemicals Co. Inc. Urease (020-83242, Kishida Chemical, Osaka, Japan), purified from jack bean meal and with urease activity of 2950 U/g, was used in the bio-catalytic dissociation of urea. Poorly graded silica sand #6 with e_max_, e_min_, C_u_, and specific gravities of 0.899, 0.549, 1.55, and 2.653, respectively, was used in this work. The grain size distribution curve of the sand utilized in this work is shown in Figure 1.

The expected reactions to obtain precipitated carbonate ions in the presence of calcium ions (Ca^2+^) and magnesium ions (Mg^2+^) supplied in the solution are shown in Eqs. 1–5.

(1) $$\text{CO}{\left(\text{N}{\text{H}}_{2}\right)}_{2}+2{\text{H}}_{2}\text{O}\to 2\text{N}{\text{H}}_{4}^{+}+\text{C}{\text{O}}_{3}^{2-}$$

(2) $$\text{CaC}{\text{l}}_{2}\to \text{C}{\text{a}}_{2}^{+}+2\text{C}{\text{l}}^{-}$$

(3) $$\text{MgC}{\text{l}}_{2}\to \text{M}{\text{g}}_{2}^{+}+2\text{C}{\text{l}}^{-}$$

(4) $${\text{Ca}}_{2}^{+}+{\text{CO}}_{3}^{2-}\to {\text{CaCO}}_{\text{3}}$$

(5) $$\text{M}{\text{g}}_{2}^{+}+\text{C}{\text{O}}_{3}^{2-}\to \text{MgC}{\text{O}}_{3}$$

### Test-Tube Experiments

In this work, the precipitation of carbonate was evaluated directly in transparent test tubes. The magnesium was substituted in the CaCl_2_–urea solutions whose concentrations varied from 10 to 50% of the initial concentration of CaCl_2_ (i.e., 0.5 mol/L). The total concentration of CaCl_2_–MgCl_2_ was fixed at 0.5 mol/L. One gram per liter of urease was used to dissociate 0.5 mol/L of urea. The experimental conditions for the precipitation tests are listed in Table 1. Urease powder was mixed with distilled water, stirred for 2 min, and then filtered using filter paper (pore size of 11 μm) to remove the undissolved particles. Combinations of CaCl_2_–MgCl_2_–urea and the purified urease were mixed thoroughly to make a total solution volume of 30 mL and allowed to react until the system reached the steady state. During the entire 6-day curing time, the test tubes were kept in a box without shaking. Note that the solutions were always mixed and the samples were always cured at a room temperature of 20°C. After 24 h, the solutions were filtered through the filter paper (pore size of 11 μm). The particles deposited on the filter paper and the particles remaining in the tubes were dried at 60°C for 24 h, and the total amount of precipitated materials was evaluated by combining the precipitated materials deposited in the test tubes with the materials remaining on the filter paper. The precipitation ratio, which is the ratio of the actual mass of the precipitated materials to the theoretical mass of the maximum precipitation of CaCO_3_, was obtained. The theoretical mass of CaCO_3_ (g) was evaluated as *C*·*V*·*M*, where *C* and *V* represent the concentration of the solution in moles per liter and the volume of the solution in liters, respectively, and *M* is the molar mass of CaCO_3_ of 100.087 g/mol. The actual mass is the mass of the precipitated materials in grams evaluated from the tests. The evaluation of the precipitated mass was conducted every 24 h until the system reached the steady state. Two identical tests were performed for each condition to check the reproducibility.

**Table 1:** **Experimental conditions for precipitation tests**.

Sample case	Concentration of CaCl_2_		Concentration of MgCl_2_	
	Ratio (%)	(mol/L)	Ratio (%)	(mol/L)
C0	100	0.50	0	0.00
C1	90	0.45	10	0.05
C2	80	0.40	20	0.10
C3	70	0.35	30	0.15
C4	60	0.30	40	0.20
C5	50	0.25	50	0.25

Test-tube experiments were conducted to examine the effect of magnesium on the rate of urea hydrolysis in the presence of urease. The measurement of the evolution of pH with time might indirectly define the rates and the magnitude of urea dissociation accelerated by the urease (Yasuhara et al., 2012). The evolving pH was measured using a pH meter 0, 1, 2, 3, 6, 7, 8, 9, and 10 h after mixing. The amount and the characteristics of the precipitated materials obtained from the test-tube experiments, corresponding to the different ratios of CaCl_2_–MgCl_2_, were also evaluated in this work. XRD and SEM analyses of the precipitated carbonate were conducted to analyze and to examine the mineralogical substances.

### Unconfined Compressive Tests

Unconfined compressive strength tests were carried out to evaluate the improvement in stiffness and strength of the treated sand specimens. PVC cylinders (5 cm in diameter and 10 cm in height) were used to prepare the sand samples. The fixed volume of the solution was injected into each prepared sand specimen. The injected volume was controlled by the number of pore volumes, one pore volume being ~75 mL. The experimental conditions for the PVC cylinder tests are listed in Table 2.

**Table 2:** **Experimental conditions for PVC cylinder tests**.

Sample case (–)	Number of pore volumes (times)	Maximum precipitated^a^	
		Mass (g)	Ratio (%)
U1	1	3.75	1.25
U2	2	7.50	2.50
U3	3	11.25	3.75
U4	4	15.00	5.00
U6	6	22.50	7.50
U8	8	30.00	10.00

*^a^Calcite content in 300 g of treated sand*.

First, 300 g of dry silica sand were poured into the PVC cylinders to obtain a relative density of 50%. Second, 75 mL (i.e., one pore volume) of the optimum grout solution, obtained from the test-tube experiments, were poured into the PVC cylinders from the top. The curing time of the PVC cylinder tests was determined by observing the precipitation tests. After the curing time, the treated specimens were removed from the PVC cylinders. The surface of the treated samples was flattened before the UCS tests were conducted. Two tests were performed for each condition to check the reproducibility. The UCS tests were conducted under wet conditions to avoid any unexpected precipitation that may occur when samples are intentionally dried out.

The acid leaching method was used to evaluate the amount of precipitated calcite (Yasuhara et al., 2011, 2012; Neupane et al., 2013). In this process, the treated sand was washed with distilled water to dissolve the salt material and then dried in an oven at a temperature of 100°C for 24 h. The dried sand was weighed and washed with 0.1 mol/L of HCl several times until air bubbles no longer appeared. Filter paper (pore size of 11 μm) was used to minimize the lost mass of sand during the washing process. The sand was dried again, and the final weight was taken. The dry weight lost during the acid leaching was evaluated and assumed to be the weight of the precipitated carbonate. The reaction taking place is expressed by Eq. 6.

(6) $${\text{CaCO}}_{\text{3}}+2\text{HCl}\to {\text{CaCl}}_{\text{2}}+{\text{CO}}_{\text{2}}+{\text{H}}_{2}\text{O}$$

**Figure 2:**
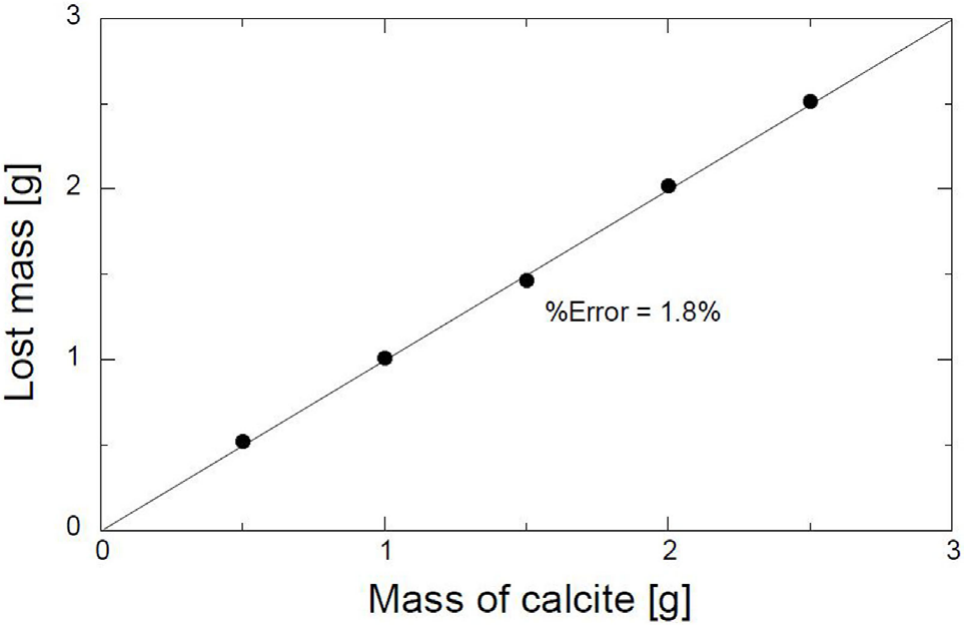
**Evaluation curve for acid leaching method of calcite quantification**.

Similar tests were conducted to eliminate any errors that might occur due to the loss of sand during washing. The experimental conditions are shown in Table 3. Various masses of CaCO_3_ were also evaluated under the same conditions; refer to Table 3. The relation between the mass of CaCO_3_ and the weight lost during the acid leaching is shown in Figure 2. The average percentage error was calculated to be 1.8%. The percentage error is the ratio of the difference between the mass of CaCO_3_ and the lost mass. The minimum percentage error indicates that the acid leaching method was reasonable.

**Table 3:** **Experimental conditions for evaluation of acid leaching test results**.

Sample name (–)	Mass of CaCO_3_ (g)	Mass of sand (g)	Total mass (g)	Lost mass (g)	Percentage error (%)
Cal1	0.5	49.5	50	0.52	4.0
Cal2	1.0	49.0	50	1.01	1.0
Cal3	1.5	48.5	50	1.47	2.0
Cal4	2.0	48.0	50	2.02	1.0
Cal5	2.5	47.5	50	2.52	0.8

## Results and Discussion

**Figure 3:**
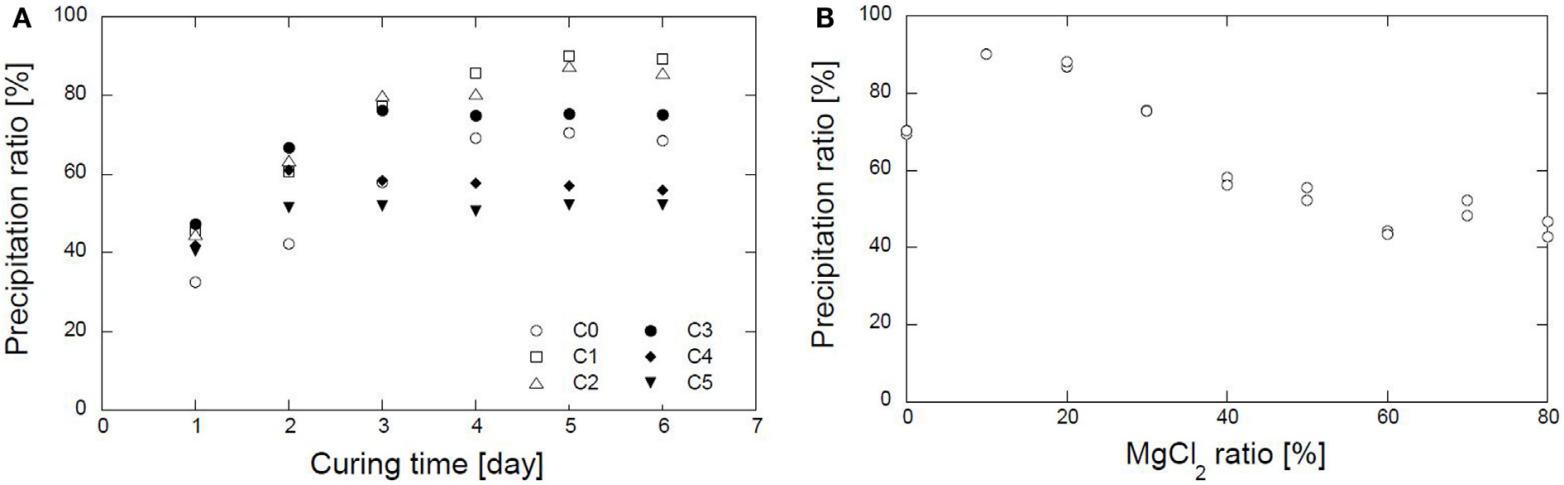
**Test-tube experiment results. (A)**. Precipitation ratio results in several curing time. **(B)** Relation between magnesium ratio and carbonate precipitation ratio.

The precipitation ratios of various combinations of CaCl_2_–MgCl_2_ with 0.5 mol/L urea and 1.0 g/L urease for several curing times were evaluated. Precipitation approached the steady-state condition after 5 days (Figure 3A). A summary of the precipitation ratios after 5 days of curing is shown in Figure 3B. As is apparent in the figure, the combinations that contain magnesium of 10, 20, and 30% have higher precipitation ratios than those without magnesium. The maximum precipitation ratio without magnesium was roughly 70%. The precipitation ratio increased rapidly and approached the maximum, i.e., 90%, when 10 and 20% of magnesium were used. Subsequently, it decreased gradually as the magnesium ratios increased even further. It is shown that the substitution of a small amount of magnesium brings about a significant improvement in the precipitation ratio. The improvement may be due to the Mg^2+^ ions employed in the reaction. The presence of Mg^2+^ ions may promote the formation of aragonite, with a specific gravity higher than calcite (Oomori and Kitano, 1985; Boyd, 2012). Aragonite with Mohr hardness stronger than calcite can form in the presence of magnesium ions and pH <11 (Tai and Chen, 1998). By contrast, the substitution of 40–80% magnesium causes a gradual decrease in the precipitation ratio, resulting in a reduction in the amount of carbonate precipitated.

**Figure 4:**
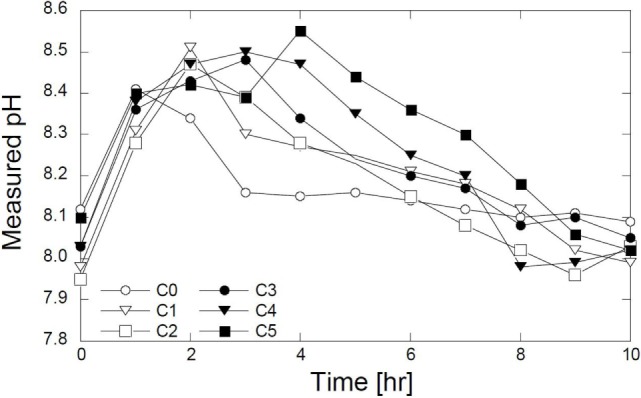
**Evolution of pH with time**.

The results of pH measurements are shown in Figure 4. All the measured pH levels increased rapidly after 1 h. For the C0, the pH decreased gradually after 1 h and then approached the steady state after 3 h. In C1, C2, and C3, the decrease in pH started after 2 h and approached constant values after 8 h. In C4 and C5, the pH decreased after 4 h. The increase in elapsed time indicates that the magnesium should influence the reaction time – the presence of the Mg^2+^ ions delays the reaction. The magnesium could be used as the delaying agent in calcite precipitation. In the C0, $\text{C}{\text{O}}_{3}^{2-}$ ions directly bind with Ca^2+^ ions. As the magnesium was substituted, the $\text{C}{\text{O}}_{3}^{2-}$ ions were likely to have bound with the Ca^2+^ and Mg^2+^ ions, which caused a delay in the calcite formation.

**Figure 5:**
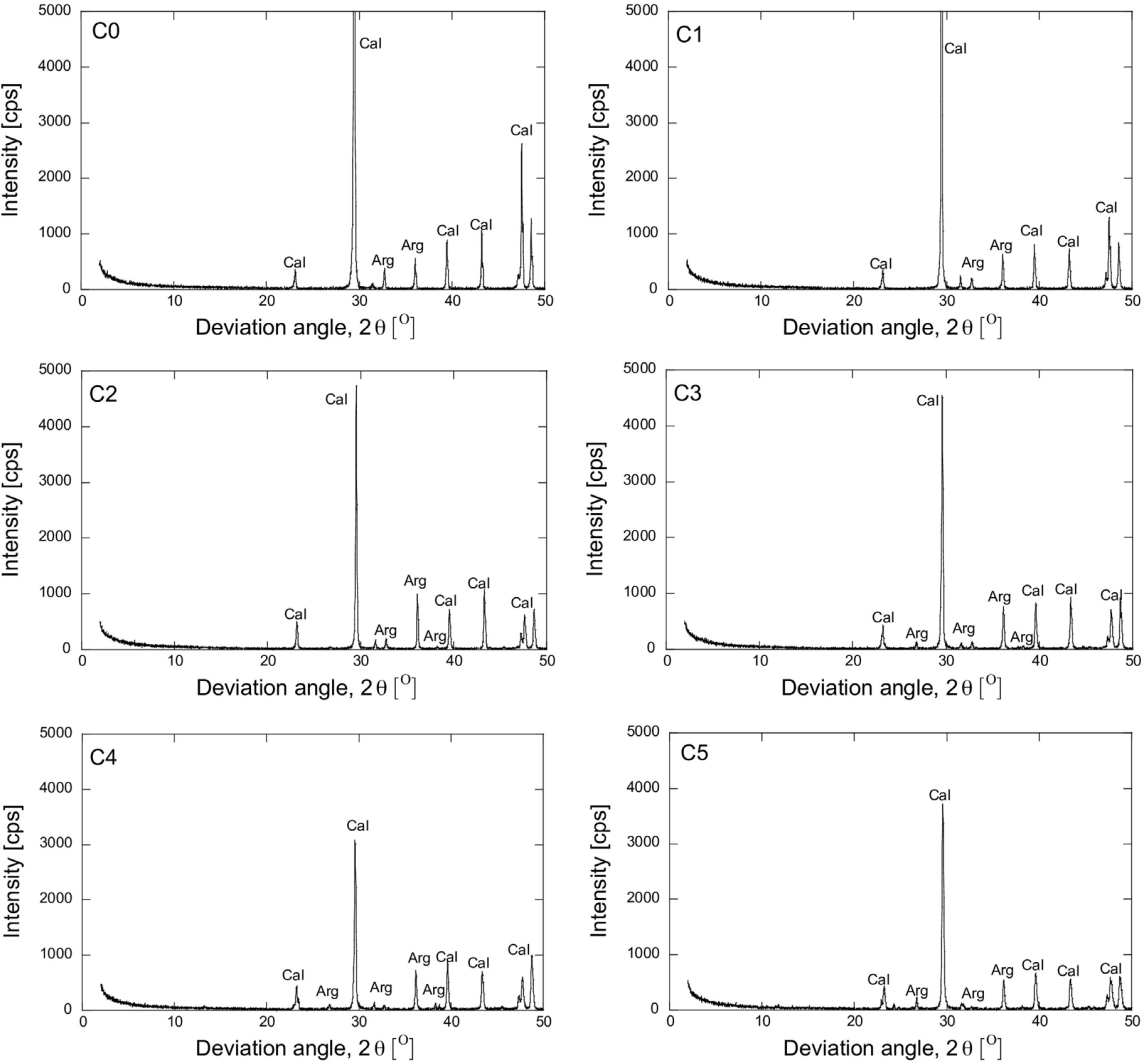
**X-ray diffraction results of precipitated material**.

The addition of magnesium also affected the shape of the precipitated materials. XRD and SEM analyses were conducted to evaluate the effect of magnesium. The XRD results in Figure 5 show the impact of magnesium on the crystalline material. The main material was calcite. The substitution of magnesium decreased the intensity of the calcite peak and promoted the aragonite peaks. As 10% of magnesium was substituted, the intensity of the primary (2θ = 29.75) and the secondary (2θ = 47.54) peaks of calcite decreased significantly. Subsequently, the main peak of calcite (2θ = 29.75) decreased gradually as the magnesium ratio increased even further. The aragonite peaks are clearly shown when the substitution of more than 20% magnesium was conducted. Referring to the Debye–Scherrer equation, the crystal size could be examined from the relationship between the intensity and the deviation angle from the XRD results, given by Eq. 7 (Monshi, 2012).

(7) $$D=\frac{\text{Kλ}}{\text{βcosθ}}$$

**Figure 6:**
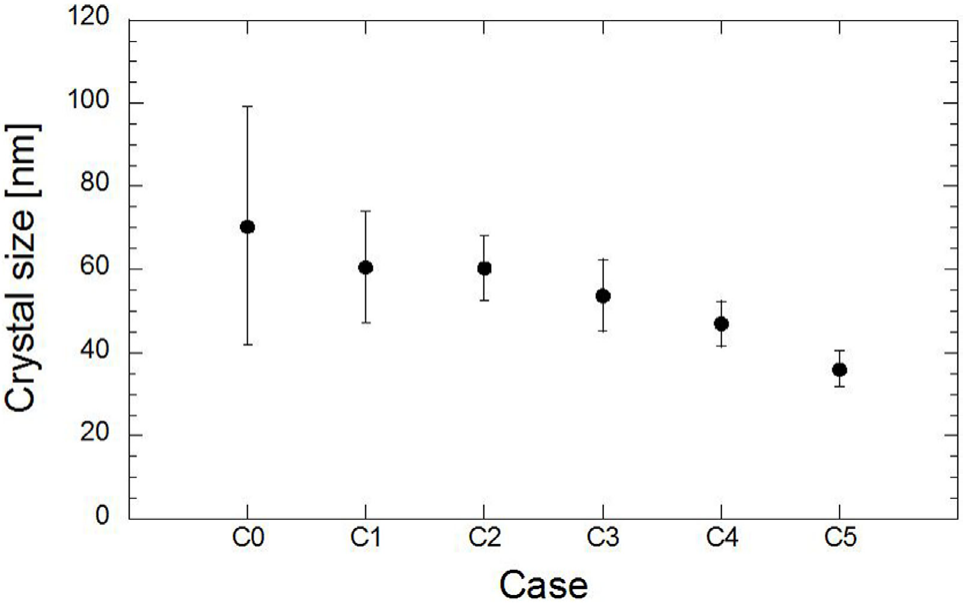
**Effect of magnesium on evolution of crystal size**.

*D* is the crystal size in nanometer (nm), λ is the X-ray wavelength, β is the full width at half maximum (FWHM) in radians, and *K* is a constant related to the crystallite shape, generally taken as 0.89 for spherical crystals with cubic unit cells (Monshi, 2012). The value for β in the 2θ axis of the diffraction profile must be in radians. The effect of the substitution of magnesium on the size of the crystal material is shown in Figure 6. The additional magnesium caused a gradual decrease in the crystal size as the magnesium ratio further increased. The substitution of 10 and 20% of magnesium reduced the crystal size to 14% of the initial size, while the substitution of 50% magnesium reduced the crystal size to half of the initial size.

**Figure 7:**
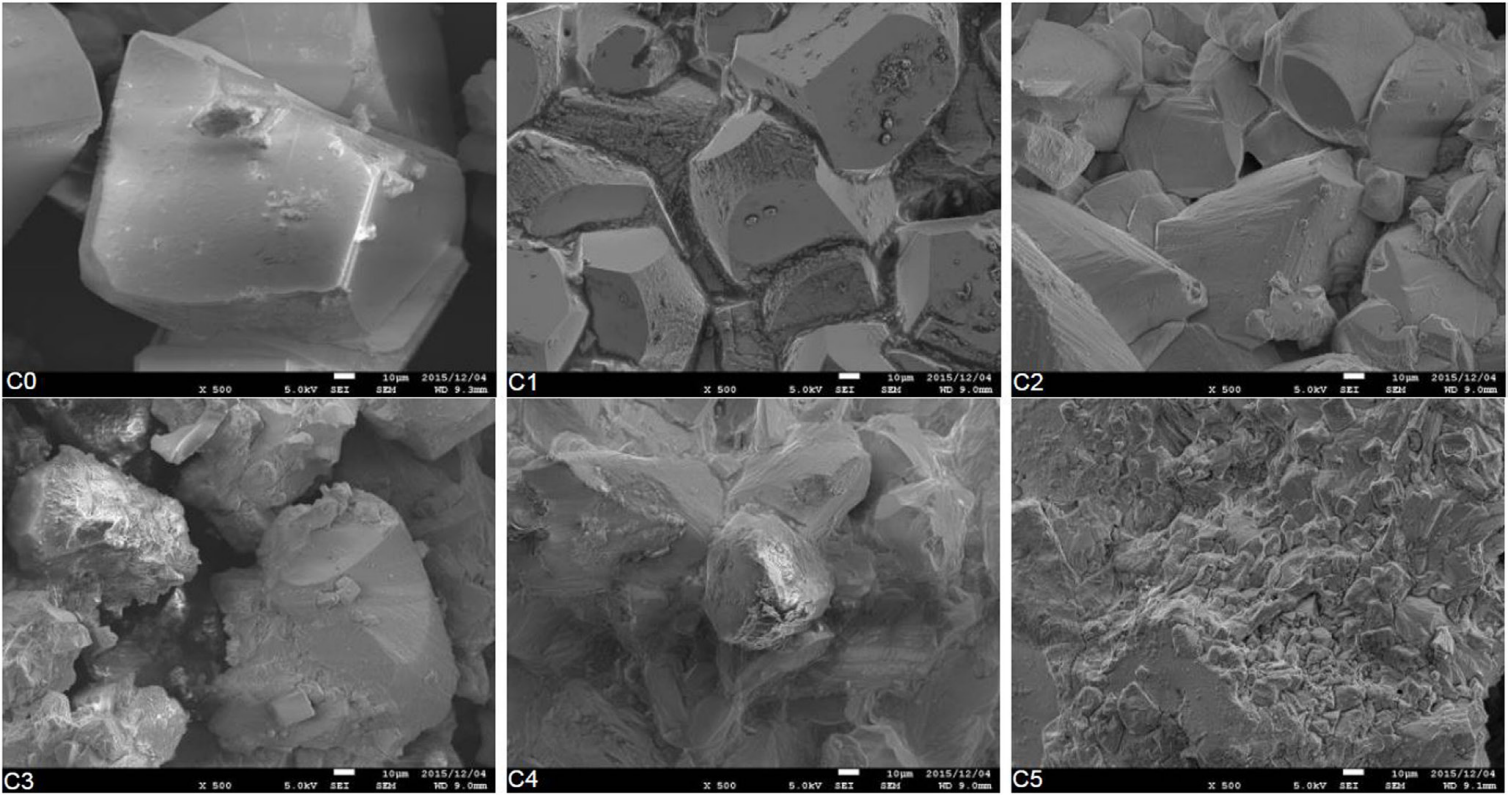
**The evolution of crystal shape as the effect of the substitution of magnesium**.

The evolution of the crystal shapes obtained from the SEM analysis is shown in Figure 7. The C0 is the crystal structure image of calcite without the precipitation of magnesium. In such a case, the carbonation process may cause the formation of rhombohedral calcite. The substitution of magnesium modified the structure of the crystal. The rhombohedral pattern of the calcite could be observed until the additional of 20% magnesium (i.e., C2). The substitutions of magnesium decreased the amount of Ca^2+^ ions. The lower concentration of Ca^2+^ ions promoted the amorphous structure of the precipitated calcite minerals.

**Figure 8:**
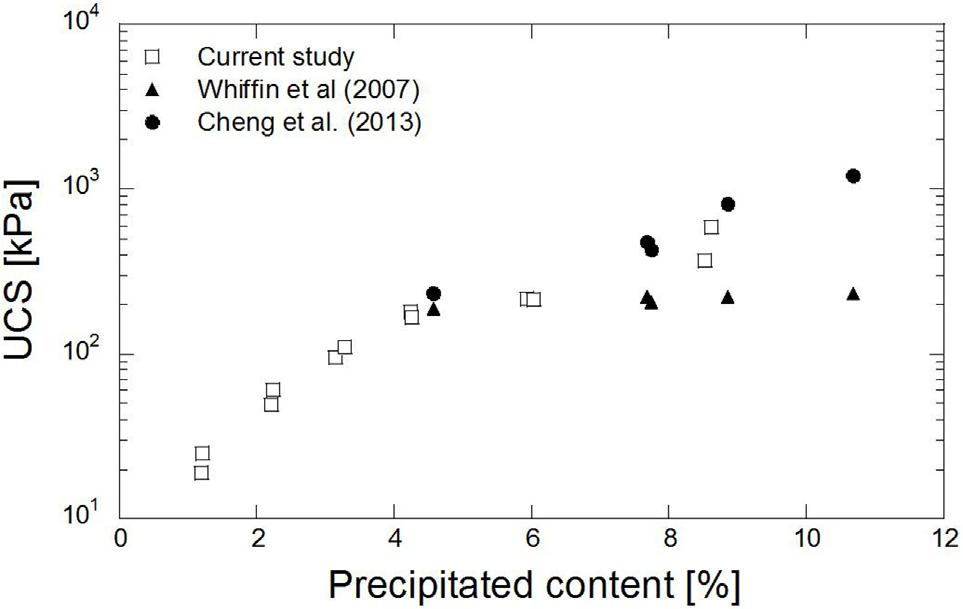
**Relationship between precipitated carbonate content and UCS tests**.

The relation between the content of the precipitated carbonate and UCS is depicted in Figure 8. The maximum UCS of 0.6 MPa was obtained from the treated sample containing 8% precipitated carbonate. In comparison to the previous study, which addressed calcite precipitation without magnesium (Whiffin et al., 2007), the strength obtained in this study was roughly 40% higher for the same carbonate content. Cheng et al. (2013) obtained the UCS up to 0.8 MPa from the 9% precipitated content. A similar trend to that seen in the previous study was obtained. The existence of aragonite may contribute to the increase in strength of the treated sand. Aragonite is a more compact structure of carbonate groups. The structure and the size of carbonate may influence the strength of the treated sand. The presence of agglomeration crystals in the precipitated materials, as observed in the SEM images, may increase the adhesion of the treated sand. The precipitated carbonate in sandy soil may cause the formation of a coating over the sand grains and bridges between them and bring about the binding of the sand particles. The UCS increased gradually as the precipitated content further increased. It was possible to control the UCS of the treated specimens by adjusting the amount of precipitated materials.

## Conclusion

The efficacy of EMCP as a soil-improvement technique was evaluated. Purified urease was utilized to bio-catalyze the hydrolysis of urea to precipitate as carbonate. In this work, magnesium was newly added to optimize the rate and the amount of carbonate precipitation. In particular, the effects of magnesium chloride on the precipitated materials were examined. XRD and SEM analyses were also conducted to analyze and to examine the mineralogy composition and the microstructures of the precipitated materials.

Magnesium increased the precipitation ratio of carbonate up to 90% with the urease concentration of 1.0 g/L, and was able to be used as a delaying agent for the carbonate precipitation. The presence of magnesium changed the shape and the size of the precipitated crystals and may have resulted in the aragonite together with calcite. The presence of Mg^2+^ ions reduced the size of the precipitated crystals. The agglomeration of carbonate was generated by the substitution of a small amount of magnesium. UCS tests on the treated specimens revealed that precipitated carbonate was capable of noticeably modifying the mechanical properties of the soil. Relatively higher strength was obtained in this study – the presence of aragonite, induced by the substituted magnesium, improved the UCS of the treated specimens. The relation between the UCS of the treated sand and the amount of precipitated materials indicated that the strength could be controlled by the mass of the precipitated materials.

## Author Contributions

HP has conducted all the experiments. HY has supervised this work and suggested how to conduct the experiments. NK has suggested how to proceed this work and partly conducted the experiments. DN has suggested how to proceed this work and partly conducted the experiments. CL has suggested how to proceed this work and partly analyzed the data.

## Conflict of Interest Statement

The authors declare that the research was conducted in the absence of any commercial or financial relationships that could be construed as a potential conflict of interest.
